# Clinical review of 24–35 year olds conceived with and without in vitro fertilization: study protocol

**DOI:** 10.1186/s12978-017-0377-3

**Published:** 2017-09-20

**Authors:** Sharon Lewis, Joanne Kennedy, David Burgner, Robert McLachlan, Sarath Ranganathan, Karin Hammarberg, Richard Saffery, David J. Amor, Michael M. H. Cheung, Lex W. Doyle, Markus Juonala, Susan Donath, John McBain, Jane Halliday

**Affiliations:** 1 Murdoch Children’s Research Institute, The Royal Children’s Hospital, Parkville, 3052 Australia; 2 Department of Paediatrics, University of Melbourne, Melbourne, Australia; 3 Department of Paediatrics, Monash University, Melbourne, Australia; 4 Hudson Institute of Medical Research, Monash University, Clayton, 3168 Australia; 5 Monash IVF Group, Richmond, 3121 Australia; 6 Department of Respiratory and Sleep Medicine, The Royal Children’s Hospital, Melbourne, Australia; 7 Victorian Assisted Reproductive Treatment Authority, Melbourne, Australia; 8 The Royal Children’s Hospital, Melbourne, Australia; 9 Department of Cardiology, The Royal Children’s Hospital, Melbourne, Australia; 10 Research Office, Royal Women’s Hospital, Melbourne, Australia; 11 Department of Obstetrics and Gynaecology, University of Melbourne, Melbourne, Australia; 12 Department of Internal Medicine, University of Turku and Division of Medicine Turku University Hospital, Turku, Finland; 13 Reproductive Services, Royal Women’s Hospital, Melbourne, Australia

**Keywords:** Assisted reproductive technologies, In vitro fertilization, Adults, Cardiovascular health, Respiratory health, Metabolic health

## Abstract

**Background:**

Children conceived by assisted reproductive technologies (ART) currently comprise 4% of Australian births. The manipulation of biological parameters related to fertilization and implantation are integral to successful ART but potentially pose a risk to the longer-term health of the offspring. There is consensus that many common adult health problems (particularly cardiovascular, metabolic and respiratory conditions) have their origins in early life, possibly before birth, and that risk trajectories track through childhood until clinical disease manifests in adulthood. Early life epigenetic variation may play a role in this process. However little is known about the long-term health of individuals conceived by ART. In a previous study, based on telephone-interviews, we found that young adults conceived by in vitro fertilization (IVF) had significantly more maternal reported atopic respiratory, endocrine, nutritional, and metabolic conditions than non-IVF conceived matched controls. Here we outline the protocol for a follow-up biomedical assessment of this cohort and a questionnaire to obtain information on potential confounders.

**Methods:**

We are conducting a clinical review of an existing, well characterised cohort comprising 547 IVF-conceived adults and 549 matched controls. We are measuring cardiovascular intermediate phenotypes, metabolic parameters and respiratory function, complemented by epigenome-wide DNA methylation analysis. A pilot study demonstrated the feasibility of our proposed protocol and its acceptability to participants. Participants attend a 2–3 h clinical assessment and complete a study-specific online questionnaire. Measurements include: 1) cardiovascular phenotypes: carotid artery intima-media thickness and distensibility, retinal vascular calibre, resting blood pressure, pulse wave velocity and pulse wave analysis; 2) respiratory function: spirometry, plethysmography, multiple breath washout; 3) auxology: height, weight, waist circumference, bio-impedance. Blood is collected for 4) biomarkers of cardiometabolic profile including inflammatory markers and 5) epigenetic analysis.

**Discussion:**

Recruitment for this clinical review is challenging as many of the participants have moved to regional, interstate or international locations. Additionally, many female participants are pregnant or breastfeeding, and are therefore ineligible. Nevertheless, comprehensive strategies have been developed to optimize recruitment. Given the increasing use of IVF and related technologies, the potential long-term consequences for risk of common adult diseases is an important clinical and public health issue.

## Plain English summary

There is a widely-held belief that many common adult onset disorders (particularly heart and lung related diseases) begin in early life, possibly even before birth. Children born following the use of assisted reproductive technologies (ART) such as in vitro fertilization (IVF) might have a higher risk of such disorders due to the handling of eggs, sperm and the resulting embryos in the laboratory. However there are very few studies on adults born following ART, and those that have been done are small in size. We are recruiting participants for a clinical study of heart and lung health from two groups of people who participated in an earlier study: 547 born following IVF and 549 age- and sex-matched non-IVF conceived adults aged 24–35 years. We are measuring their blood pressure, heart rate, height, weight, body mass index and body fat content, undertaking an ultrasound scan of the blood vessels of the neck and completing tests to look at the stiffness of blood vessels, and the function of the respiratory system (lungs). We are also taking a photo of the blood vessels in the back of their eye. Australia, as elsewhere, has an increasing proportion of infants who have been conceived by ART (currently 4% but rising). By examining the health of IVF-conceived individuals in adulthood, we will improve understanding about long-term health effects of ART and generate important and unique data that may have wide biological and clinical implications.

## Background

Many common adult health problems, particularly cardiovascular, metabolic and respiratory conditions, have their origins in early life, possibly even before birth [1]. Children born following the use of assisted reproductive technologies (ART) may be at increased risk of adult health problems, in part because of the laboratory techniques used to create embryos and enable pregnancy [2, 3]. In addition to the possible risks posed by these techniques, numerous studies have demonstrated adverse perinatal outcomes after ART [4–7], which themselves can have consequences for adult heath. For instance, low birth weight, which is more common after ART than spontaneous conception, has been associated with metabolic disturbances in childhood and adulthood [8], ischaemic heart disease [9] and diminished lung function [10].

The laboratory techniques used in ART and the adverse perinatal outcomes associated with ART may increase the risk of non-communicable diseases in adulthood. Few studies have yet been able to investigate the prevalence of non-communicable diseases in large cohorts of ART-conceived young adults [2], primarily due to the relatively short time since ART was developed. The limited existing evidence suggest a higher risk of cardiometabolic problems in ART-conceived than non-ART conceived adults [3, 11]. For example systolic and diastolic blood pressure was higher in cohorts of ART-conceived offspring compared with controls, after controlling for gestational age and birth weight, while fasting glucose was higher in the ART conceived pubertal subgroup of one of these cohorts [12–15].

More data exist for younger age groups. Systemic and pulmonary vascular dysfunction has been demonstrated in ART-conceived children [16], while there have been inconsistent findings regarding their growth and body fat composition. Some studies have shown a more central, peripheral and total adiposity distribution in ART-conceived children compared with controls [17–19], but others have found no increase in BMI or body size [15, 20].

In a previous study, using telephone-interviews, we compared the health and wellbeing of 547 singleton young adults (aged 18–28 years) born following in vitro fertilization (IVF) with 549 matched controls [21, 22]. Maternally reported data indicated that IVF-conceived singletons had a significant increase in respiratory atopy relative to matched controls after adjusting for confounders [21]. Interestingly, a similar association has been identified in other studies of children conceived using ART [23, 24].

Potential ‘programming’ of adult onset risk by early life exposures such as ART may be due to an aberrant epigenetic profile established in utero, a theory supported by studies demonstrating DNA methylation variation in ART conceived offspring [25, 26]. Such changes have been associated with an increased prevalence of rare imprinting disorders such as Beckwith-Wiedemann syndrome [27]. It is postulated that the very early periconceptional period is particularly sensitive to environmental influences, such as the hormone milieu created by ovarian stimulation or in vitro culture used in IVF [11, 28]. However, available data are circumstantial, limited and often contradictory.

In the face of increasing use of ART and the growing population of ART-conceived young adults, there is a need to establish whether adverse health outcomes are associated with ART. Further, given mounting evidence for a role of epigenetic variation in a range of complex disorders, the potential for epigenetic disruption by ART warrants scrutiny. This protocol outlines a study to undertake a clinical review of the largest reported cohort of IVF-conceived adults worldwide and spontaneously conceived controls, to ascertain whether conception by IVF is associated with well-established intermediate risk profiles for cardiovascular, metabolic and respiratory disease in adulthood.

## Methods/design

### Aims

Our primary objective is to characterise the cardio-respiratory phenotype of an existing cohort of young adults (now aged 24–35 years) conceived by IVF, and investigate underlying biological mechanisms, including epigenetic (DNA methylation) profile.

The main aims are:

1. To investigate the long-term health consequences of assisted reproduction by comparing the cardiovascular, metabolic and respiratory status of adults conceived by IVF with age- and sex-matched spontaneously conceived controls, using standardised, validated non-invasive clinical assessment tools;
2. To identify the epigenetic consequences of IVF conception by:
  a) comparing the DNA methylation profile in blood spots collected around the time of birth of the IVF-conceived adults with that of controls;
  b) investigating whether significant IVF-associated methylation variants found at birth are still present in adulthood.

### Study design

Prospective cohort.

### Setting

The study setting is the Murdoch Children’s Research Institute (MCRI), located at The Royal Children’s Hospital in Melbourne, Australia.

### Study timeline

A pilot study was conducted in 2014 to determine the feasibility of re-contacting and recruiting the previously studied cohorts for clinical review, as well as testing the implementation of the planned measurement protocol and processes. Preparation for the main study commenced in 2014 with funding and ethics applications. Recruitment and data collection for the main study is being undertaken in 2016 and 2017 with data analysis to commence late 2017.

### Ethics approval

Ethics approval was granted by Melbourne IVF HREC (Project 26/13) for the pilot study and The Royal Children’s Hospital HREC (Project 33163) for both the pilot and main study.

### Eligibility criteria

Potential participants are the IVF-conceived (IVF) and non IVF-conceived (control) young adults who participated in the previous study [21, 22] and who consented to be contacted for further research. Participants are required to be able to attend MCRI for all aspects of the clinical review.

### Exclusion criteria

Women who are pregnant or breastfeeding.

### Recruitment procedures

Participants from the previous study are selected at random, grouped into batches, and sent a letter of invitation, along with a copy of the patient information sheet and consent forms (PICF) and a reply paid envelope. The letter and PICF provide a detailed explanation of the study, what participation involves, and instructions on how to provide consent. If a response is not received after three weeks, a reminder slip is sent informing participants that there is still time to participate. If there is still no response three weeks later, a phone call is made to the contact number on record. This allows us to ascertain if the individual has received the invitation and to answer any questions they may have about the study. For those that have not received the invitation, their postal address is updated and the study documents posted again.

**Fig. 1:**
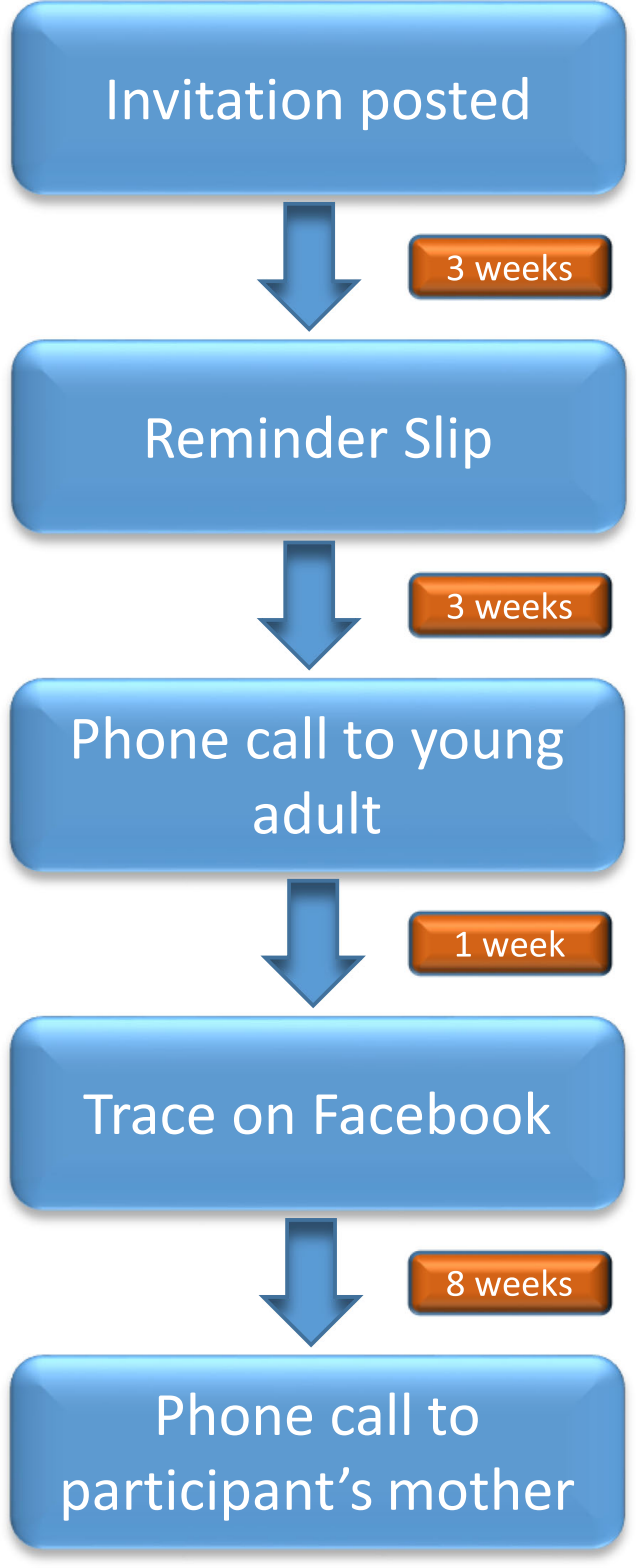
Flowchart of follow-up of non-responders

If the telephone number we have is invalid, we attempt to trace individuals via social media (Facebook). If this fails we contact the individual’s mother who participated in the first study, using previously recorded information. Depending on the mother’s preference, the study documents are either sent to the mother to pass on to her daughter or son, or sent directly to the potential participant using the updated contact details provided by the mother. Figure 1 shows the flow of events in the follow up of non-responders.

Once a signed consent form is received, a member of the research team contacts the participant to arrange a time for the clinical review and to email the questionnaire. All clinical reviews are conducted at the Melbourne Children’s Trials Centre (MCTC), co-located at The Royal Children’s Hospital and Murdoch Children’s Research Institute. Depending on participant preference, clinical reviews can be conducted during office hours (8 am-5 pm Monday to Friday), evenings or on Saturday mornings.

### Training of clinical assessors

All clinical assessors are trained by members of the Respiratory and Cardiology Departments of The Royal Children’s Hospital, Melbourne, using their standard protocols.

### Outcomes

A summary of the outcome measures is shown in Table 1. All participants are asked to fast for at least 6 h prior to their clinical review. They are asked not to have anything to eat and only water to drink from the time specified by the research team.

**Table 1:** Study outcomes

	Outcomes	*Measures*
Clinical	Cardiovascular	
	Structure	Carotid artery intima-media thickness, retinal vessel photography
	﻿Function	Resting blood pressure, pulse wave velocity, augmentation index, carotid artery distensibility
	Auxology	Height, weight, waist and hip circumference, bio-impedance
	Respiratory function	Spirometry, plethysmography, multiple breath washout
Biomarkers	Cardiometabolic profile	Standard lipid profile and free fatty acids, fasting blood glucose, fasting blood insulin
	Inflammatory plasma marker	High sensitivity C-reactive protein
	Epigenetics	Genome-wide DNA methylation from blood derived at the time of birth (archival newborn screening cards) and at the time of the clinical assessment (adulthood). Gene expression from blood collected in adulthood.

### Measures

#### Cardiovascular assessments

(i) ***Carotid intima-media thickness*** **(**cIMT*)*: Images are acquired using a GE Vivid ultrasound machine with a high frequency broadband linear probe and simultaneous ECG monitoring, using standardised techniques in accordance with recommendations of the American Society of Echocardiography and Mannheim Consensus statements [29, 30]. Magnified digital images of the distal 10 mm of the right common carotid artery is acquired, and the intima-media thickness is measured at five sites at end-diastole on the R wave of the ECG. In order to measure distensibility of the vessel, the maximal and minimal diameters of the vessel are measured. The intima-media thickness of the far wall of the carotid artery and arterial diameters is measured off-line using automated edge-detection software (CarotidAnalyzer, Medical Imaging Applications LLC).
(ii) ***Retinal vessel images*** are captured using a high-resolution camera without a mydriatic. A photograph (at least 30°) of the fundus (the back of the eye) is taken of both eyes, centred on the optic disc. Digital retinal images will be assessed with semi-automated software for the measurement of retinal arterioles and venules. Based on the six biggest arterioles and veins, these individual measurements will be summarised as the Central Retinal Artery or Vein Equivalent.
(iii) ***Blood pressure (BP)***: the mean is calculated of two resting seated measurements taken 5 min apart in the non-dominant arm, using a Welch Allyn ProBP3400 automated sphygmomanometer.
(iv) ***Pulse wave velocity (PWV)***: Arterial stiffness is assessed by central and peripheral PWV and pressure waveform analysis using the SphygmoCor® system (ref). A blood pressure cuff on the left upper arm acquires pressure waveforms from the left brachial artery, yielding the augmentation index and, in conjunction with a pressure tonometer, pulse wave velocity.
(v) ***Carotid artery distensibility***: Arterial distensibility and elasticity are measured automatically by Carotid Analyzer from at least three maximum and minimum vessel diameter frame pairs.

#### Auxology assessments

Auxology measures (height measured using a stadiometer, weight (participants are weighed wearing light clothing and without shoes), and waist circumference) are recorded and age and sex adjusted body mass index z-score calculated (height [m]/weight [kg]^2^) in accordance with the US Centers for Disease Control growth reference charts [31]. These measures will be used as part of a metabolic syndrome (MetS) assessment which will be defined as per international guidelines and in previous studies [32, 33]. MetS will be diagnosed if the participant has at least three of the following five metabolic risk factors: *1*) large waistline (waist circumference ≥ 102 cm for males and ≥88 cm for females), *2*) high triglyceride levels (triglycerides ≥1.7 mmol/L (≥150 mg/dL)) or specific treatment for elevated triglyceride levels, *3*) Low HDL cholesterol (<1.0 mmol/L (<40 mg/dL) in males or <1.3 mmol/L (<50 mg/dL) in females) or specific treatment for low HDL cholesterol levels, *4*) high blood pressure (≥130/85 mmHg) or treatment of previously diagnosed hypertension, and *5*) high fasting plasma glucose (≥5.6 mmol/L (≥100 mg/dL)) or specific drug treatment of elevated glucose). Adiposity is measured using bio-impedance scales to calculate body composition including total fat mass, lean mass and visceral fat mass.

#### Respiratory function assessment

Respiratory function is measured in accordance with guidelines of the American Thoracic Society [34]. The following variables are measured: forced expired volume in 1 s (FEV_1_), expiratory flow at 75% (FEF_75_), 50% (FEF_50_) and 25% (FEF_25_) of vital capacity, and forced mid-expiratory flow (FEF_25–75_). Lung volumes include: forced vital capacity (FVC) and using plethysmography, total lung capacity (TLC), functional residual capacity (FRC) and residual volume (RV).

As standard spirometry may be relatively insensitive to mild early lung function changes associated with inhomogeneous lung changes, we are also using multiple breath nitrogen washout (MBW) to assess lung function according to the joint recommendations of the American and European Thoracic Societies [35]. The following variables are measured: lung clearance index (LCI), FRC and the first and second moment ratios (M1 and M2).

#### Biomarkers

One 9 ml EDTA and two 2.5 ml serum gel fasting blood samples and a cheek (buccal epithelial) swab are collected from all consenting participants following clinical assessment. The serum gel blood samples are processed by the Laboratory Services at The Royal Children’s Hospital, Melbourne who measure the standard lipid profile, fasting blood glucose, and fasting blood insulin levels. The EDTA blood sample is processed by the Melbourne Children’s Bioresource Centre (MCBC), located within the MCRI, for storage of plasma and viable peripheral blood mononuclear cells and extraction of DNA. All samples are frozen at -80 °C within 2 h of collection. In order to obtain DNA around the time of birth, participant newborn screening cards (Guthrie spots) will be retrieved from the Victorian population-based archive under the custodianship of Victorian Clinical Genetics Services. One of 4 (total) 8 mm diameter blood spots will be used for isolation of genomic DNA.

#### Epigenetic (DNA methylation) analysis

An epigenome-wide association study (EWAS) will be undertaken on newborn blood-spot derived genomic DNA isolated from retrieved newborn screening cards. Genomic DNA and methylation profiling will be carried out on all IVF participants and controls for whom birth blood can be identified, using a previously validated approach [36]. DNA will be isolated from a single 8 mm blood spot using an in-house modified protocol and the Zymo ZR DNA-card extraction kit (Zymo Research Corporation, Irvine, CA). Methylation profiling will be carried out using the Illumina Infinium MethylationEPIC 850 K arrays [37]. This is the EWAS platform of choice due to its low cost relative to other epigenetic measures, high genomic coverage, sensitivity and reproducibility. A total of >850,000 methylation values are obtained, spanning 99% of all genes and regulatory regions increasingly implicated in complex disease aetiology. To test the long-term stability of any ART-associated DNA methylation variants detected at birth, up to 10 genes, prioritised according to (i) effect size, (ii) demonstrated role in human health, (iii) previous evidence for disruption in association by ART, or (iv) evidence for a common pathway, will be interrogated in blood-derived DNA isolated at the time of participant’s clinical assessment. DNA methylation of these genes will be carried out using our in-house Sequenom EPITYPER analysis platform. Gene expression will subsequently be performed on any differentially methylated genes in order to ascertain likely functional consequences. This will be done using standard SYBR-green based quantitative reverse transcriptase PCR.

#### Questionnaire data

We have extensive information regarding participants’ health, development and well-being from the 150-item telephone interview they completed in the first study. At that time their mothers also completed an 80-item telephone interview about the first 18 years of participants’ lives [21, 22]. Detailed data are available on young adult physical health, quality of life, diet, psychosocial health, pubertal milestones, sexual relationships, reproductive health, education and occupation. These data will be accessed as needed in the analysis of this current clinical review study.

### Confounders

In the current study, contemporaneous data on important potential modifiers of the outcomes of interest are collected through an online questionnaire which includes questions about: a) smoking status (smoking behaviour in three year blocks from before 16 years of age to current age; b) alcohol consumption (current, based on questions developed in another study) [38] c) family history of specific manifestations of cardiovascular, metabolic and respiratory conditions including data on relationship of the affected person to the participant; d) reproductive health status including information on whether participants had ever been pregnant (or been a partner in a pregnancy), outcomes of any pregnancies, and whether they had experienced fertility difficulties and had fertility treatment., and number of biological children); e) physical exercise; f) relationship and occupational status; g) a validated measure of perceived health and quality of life (The Australian World Health Organization Quality of Life–Brief assessment) [39], and h) dietary intake (asking participants to report daily or weekly consumption of vegetables, fruit, fish, whole grains, beverages with added sugar, packaged foods and salt).

### Reporting data on clinical outcome measures

Results of the clinical review are sent to participants. If a participant’s results are outside the normal range, a referral letter for further investigation is sent to the participant to pass on to their General Practitioner.

### Sample size

We estimate that approximately 15% of the cohort will be living interstate or internationally, but that at least 450 potential participants in each group (IVF and controls) reside in Victoria. With a predicted 60% participation rate, we therefore estimate a sample size of 270 young adults in each group.

For any normally distributed variable (e.g. carotid intima-media thickness, blood pressure, cholesterol levels, lung function), a predicted sample size of 270 young adults in each group will allow us to detect a difference in means between the two groups as small as 0.25 standard deviations (effect size), with a minimum 80% power and *p* < 0.05. Other studies have found differences between ART and non-ART corresponding to considerably larger effect sizes, for example, Sherrer et al. [16] found carotid intima-media thickness (one of our measures) 410 μm +/− 30 (ART) vs 370 μm +/− 20 (non-ART), an effect size of >1.3 SD. Our study is therefore powered to detect more subtle differences than those so far found in this population. As there are only 26 sibling pairs in the potential study population, this is unlikely to affect study power.

### Statistical analysis

#### Clinical

All analyses will be done with the latest version of STATA, version 15 (StataCorp LLC, TX, USA). The distributions of continuous exposures and outcomes will be examined for normality, and will be appropriately transformed if necessary. For each of the outcome measures, unadjusted differences between IVF and control groups will be investigated using chi-square tests or Fisher’s exact test for categorical outcomes, (e.g. metabolic syndrome assessments) and two-sample *t*-tests for continuous outcomes, (e.g. blood pressure). For each outcome, differences between IVF and controls, taking into account modifiers such as sex, age, weight, height, smoking status, will be investigated using linear and logistic regression for continuous and binary outcomes, respectively. If any of the 26 potential sib pairs participate, we will account for this by fitting Generalised Estimating Equation regression models. Results will be presented as adjusted odds ratios and 95% confidence intervals.

#### Epigenetic

Methylation data will be processed in the R computing environment using minfi [40] adapted for EPIC [41]. The association between IVF and methylation at each CpG will first be assessed using robust linear regression as part of the minfi package. Differential methylation analysis within the Infinium data associated with IVF will be performed at both single CpG and region-centric levels (to increase statistical power and reduce false positives [42] using multivariate models to account for confounders, data transformation if necessary and appropriate treatments of false discovery rates.

## Discussion

In planning the study, we expected that participant recruitment would be the biggest challenge. Indeed, recruiting participants to this clinical review is proving more difficult than anticipated. First, more than 15% of potential participants who have responded to our invitation to take part in the study live in regional areas, interstate or internationally. Second, while we had assumed that the length of the clinical review (2–3 h) could be a barrier for participation, we have found that, among those who respond, it is the travel time, rather than the length of the appointment itself that influences their decision to decline our invitation. Among those who have not responded, the length of the clinical review and/or travel time may have been barriers for participation. Third, to date 8% of responding participants have reported that they are pregnant or breastfeeding rendering them ineligible to participate. Fourth, recruiting the targeted number of controls, especially male controls, is particularly challenging. Although all participants receive a letter with the results of their clinical review, and a referral letter for further investigation if required, potential controls may have less of a vested interest in the aims of the study and therefore less to gain from participating than the ART conceived young adults.

Developing comprehensive recruitment strategies, including contacting mothers of participants and tracing via social media where necessary, as well as having flexible clinical review appointment times that cover weekdays, evenings and weekends are yielding the best participation rate possible.

Depending on the final number recruited, we may need to consider the generalisability of our findings. As we have extensive questionnaire data regarding the health and well-being of all the adults from the previous study, we will be able to determine whether there is any participation bias in the current study.

## Abbreviations

ART: Assisted reproductive Technologies
BP: Blood Pressure
cIMT: Carotid intima-media thickness
EWAS: epigenome-wide association study
FEF_25_: Forced expiratory flow at 25%
FEF_50_: Forced expiratory flow at 50%
FEF_75_: Forced expiratory flow at 75%
FEV_1_: Forced expired volume in 1 s
FRC: Functional residual capacity
FVC: Forced vital capacity
IVF: in vitro fertilization
LCI: Lung Clearance index
MBW: Multiple breath nitrogen washout
MCBC: Melbourne Children’s Bioresource Centre
MCRI: Murdoch Children’s Research Institute
MCTC: Melbourne Children’s Trials Centre
MetS: Metabolic syndrome
PICF: Patient Information sheet and Consent Forms
PWV: Pulse wave velocity
RCH: Royal Children’s Hospital
RV: Residual volume
TCL: Total lung capacity
